# Bilateral Lung Transplantation in a Patient with Humoral Immune Deficiency: A Case Report with Review of the Literature

**DOI:** 10.1155/2014/910215

**Published:** 2014-10-15

**Authors:** Jocelyn R. Farmer, Caroline L. Sokol, Francisco A. Bonilla, Mandakolathur R. Murali, Richard L. Kradin, Todd L. Astor, Jolan E. Walter

**Affiliations:** ^1^Department of Medicine, Massachusetts General Hospital, Harvard Medical School, Boston, MA 02114, USA; ^2^Division of Allergy & Immunology, Massachusetts General Hospital, Harvard Medical School, Boston, MA 02114, USA; ^3^Division of Allergy & Immunology, Boston Children's Hospital, Harvard Medical School, Boston, MA 02115, USA; ^4^Department of Pathology, Massachusetts General Hospital, Harvard Medical School, Boston, MA 02114, USA; ^5^Division of Pulmonary & Critical Care Medicine, Massachusetts General Hospital, Harvard Medical School, Boston, MA 02114, USA; ^6^Pediatric Allergy & Immunology and the Center for Immunology and Inflammatory Diseases, Massachusetts General Hospital, Harvard Medical School, Boston, MA 02114, USA

## Abstract

Humoral immune deficiencies have been associated with noninfectious disease complications including autoimmune cytopenias and pulmonary disease. Herein we present a patient who underwent splenectomy for autoimmune cytopenias and subsequently was diagnosed with humoral immune deficiency in the context of recurrent infections. Immunoglobulin analysis prior to initiation of intravenous immunoglobulin (IVIG) therapy was notable for low age-matched serum levels of IgA (11 mg/dL), IgG2 (14 mg/L), and IgG4 (5 mg/L) with a preserved total level of IgG. Flow cytometry was remarkable for B cell maturation arrest at the IgM+/IgD+ stage. Selective screening for known primary immune deficiency-causing genetic defects was negative. The disease course was uniquely complicated by the development of pulmonary arteriovenous malformations (AVMs), ultimately requiring bilateral lung transplantation in 2012. This is a patient with humoral immune deficiency that became apparent only after splenectomy, which argues for routine immunologic evaluation prior to vaccination and splenectomy. Lung transplantation is a rare therapeutic endpoint and to our knowledge has never before been described in a patient with humoral immune deficiency for the indication of pulmonary AVMs.

## 1. Introduction

Humoral immune deficiencies as a group are the most common type of primary immune disorder. The underlying etiology is extremely varied, and a growing number of diverse genetic mutations have been described [1]. The defect in antibody production can range from isolated IgA deficiency, which can be clinically silent, to complete IgG deficiency, which has been associated with severe infection, requiring life-long maintenance therapy with IVIG [2]. Combined IgG subclass deficiencies have also been described. Specifically, IgA deficiency has been linked to subclass deficiencies in both IgG2 and IgG4 [3, 4]. Decreased serum levels of IgG2 and IgG4 are sufficient to confer increased susceptibility to pathogens, particularly those requiring opsonization [4]. IgG2 deficiency has also been independently associated with the development of lymphoproliferative autoimmune disease [5].

The epidemiology of humoral immune deficiency is best characterized for common variable immune deficiency (CVID) due to its prevalence in the general population. Outcomes analysis in a cohort of 473 patients with CVID [6] recently demonstrated noninfectious disease complications in 68% of patients, which included autoimmune-mediated cytopenias (thrombocytopenia (14.2%), hemolytic anemia (7%), and neutropenia (<1%)) and chronic lung disease (28.5%). In patients with chronic lung disease bronchiectasis, granuloma formation, and requirement for oxygen were frequently observed. In contrast, pulmonary AVMs were not described, and progression to lung transplant was a rare clinical endpoint (observed in only 3 out of the total 473 patients).

Here we describe a patient with humoral immune deficiencies in IgA and the IgG subclasses IgG2 and IgG4 who has been followed up at our institution over the past five years. His initial presentation was pancytopenia, for which he underwent splenectomy at the age of 18 and subsequent to which he developed recurrent infections. He was diagnosed with a “CVID-spectrum disease” elsewhere and started on IVIG therapy at the age of 20. His course was further complicated by pulmonary granulomatosis and small pulmonary AVMs, ultimately requiring bilateral lung transplantation at the age of 25. The patient is alive and continues to be followed at 21 months after transplantation.

## 2. Case Report

The patient was born prematurely at 32 weeks, yet he did not require neonatal intensive care. In childhood, he experienced recurrent acute otitis media and mild asthma; however, he was never hospitalized for a severe pulmonary disease or infection. In 2002 at the age of 15, he developed splenomegaly and cytopenias as follows: neutropenia (granulocyte antibody positive), anemia (direct antiglobulin test positive for IgG), and intermittent thrombocytopenia. In 2005 at the age of 18, he underwent splenectomy to therapeutically manage his cytopenias after a brief trial of steroids, on which he developed shingles. Subsequent to his splenectomy, the patient developed recurrent sinus (requiring hospitalization in 2007), pulmonary (requiring hospitalization in 2009—cultured positive for* H. influenzae*, methicillin-sensitive* S. aureus*, and* C. albicans*), skin (hospitalized in 2007 for abscess—cultured positive for methicillin-resistant* S. aureus*), and central nervous system (hospitalized in 2009—diagnosed with aseptic meningitis that was presumed viral) infections. In 2007 at the age of 20, the patient was diagnosed with “CVID-spectrum disease” at an outside hospital and started on IVIG infusions (40 g every four weeks). His immunologic workup was fragmented prior to 2009 when he started to follow up at the Massachusetts General Hospital for routine care.

Chart review of immunoglobulin levels in 2005, prior to initiation of IVIG therapy, was notable for low age-matched serum levels of IgA (11 mg/dL), IgG2 (14 mg/dL), and IgG4 (5 mg/dL) with normal serum levels of total IgG (1240 mg/dL) and IgM (151 mg/dL) (Table 1, “2005”). Postinitiation of IVIG therapy in 2007, trough levels of IgG2 and IgG4 normalized (Table 1, “2011”). Of note, intermittent elevations in IgG and IgM trough levels were observed between 2009 and 2012, which correlated with times of hypoalbuminemia (Table 1, comparing “2011” to “2012”). Despite preserved total IgG, the patient had pronounced clinical response to IVIG and was ultimately maintained on 40 g every three weeks to prevent breakthrough infections. Response to pneumococcal antigen prior to documented Pneumovax in 2005 was negative per report. Repeat testing was not accomplished until 2009, at which time 11/23 serotypes remained negative (1.3 mcg/mL being the accepted lower limit reference range at the Massachusetts General Hospital). Complete blood cell counts in 2009 were notable for a persistent anemia and neutropenia, with an otherwise preserved differential (Table 2). Flow cytometry of peripheral blood cells in 2009 (Table 3) demonstrated a normal number of total T cells (CD3+) and T cell subsets (CD4+ and CD8+) with CD4−/CD8− cells accounting for 5% of total lymphocytes and 6% of CD3+ cells, a finding that could suggest an increase in double-negative T cells; however, gamma delta T cells cannot be excluded in the absence of direct TCR*α* *β* staining. Flow cytometry was also notable for a normal number of total (CD19+) and memory (CD27+) B cells. However, immunoglobulin staining on the B cell surface was positive for IgM/IgD and IgM only without evidence of further class switch, suggesting a B cell maturation arrest at the IgM+/IgD+ stage. T cell function was also tested in 2009, with notable decreased proliferative response to phytohemagglutinin, pokeweed mitogen, tetanus toxoid, and Candida antigen. In terms of a malignancy workup, bone marrow biopsy in 2004 demonstrated a normocellular marrow with trilineage hematopoiesis; lymph node biopsies in 2005 and 2011 were benign, and serum electrophoresis in 2009 demonstrated minimally elevated levels of free kappa (31.9 to 95.7 mg/L—reference range < 19.4 mg/L) and lambda (40.3 to 62.6 mg/L—reference range < 26.3 mg/L) light chains with a largely preserved ratio (0.8 to 2—reference range < 1.7). Finally, the patient underwent DNA sequencing and analysis to screen for genetic mutations that have been linked to primary immune deficiencies, including those associated with hyper-IgM syndrome (*UNG*,* AICDA*,* CD40*, and* CD40LG*), CVID (*TNFRSF13B *(*TACI*)), X-linked lymphoproliferative disease (*XIAP*), and autoimmune lymphoproliferative syndrome (ALPS) (*CASP8*,* CASP10*,* FADD*,* FAS*,* FASLG*,* KRAS*,* MAGT1*, and* NRAS*). This screening was negative apart from a hemizygous G-to-A transition at position 655 that was detected in* CD40LG*, which is predicted to lead to a missense mutation and is of unknown significance for hyper-IgM syndrome type 1 [9].

In 2009, his course was complicated by severe hypoxemia (requiring 6 to 10 liters of supplemental oxygen at baseline with an arterial partial pressure of oxygen (PaO_2_) of 64 mm Hg and an arterial partial pressure of carbon dioxide (PaCO_2_) of 32 mm Hg on room air). Noncontrast computed tomography (CT) imaging of the chest demonstrated multiple nodules in the bilateral lung fields and significant mediastinal lymphadenopathy. Bronchoalveolar lavages (2009 and 2011) demonstrated rare bacteria and yeast without a consistent or predominant viral, bacterial, or fungal etiology, and cytology was without suggestion of malignancy. Quantitative ventilation/perfusion scanning demonstrated technetium-99 localization consistent with right to left pulmonary shunting, and right heart catheterization was highly suggestive of small pulmonary AVMs. Follow-up genetic testing for hereditary hemorrhagic telangiectasia (HHT) by* ACVRL1* and* ENG* gene sequencing and duplication/deletion analysis was negative. Of note,* SMAD4* was never tested; however, he underwent endoscopy and colonoscopy in 2009 and again in 2011 that was without demonstration of gastrointestinal polyps or gastrointestinal AVMs. Vascular endothelial growth factor (VEGF) levels were found to be elevated (530 pg/mL—reference range 31 to 86 pg/mL), and the patient was started on a course of suppressive doxycycline therapy.

In July of 2011, the decision was made to pursue diagnostic and therapeutic pulmonary wedge resection. Biopsies of the right upper and middle lobes demonstrated peribronchiolar lymphoid hyperplasia that was consistent with humoral immune deficiency but without clear evidence of vasculitis or AVMs. By April of 2012, he met criteria for NYHA Class III-IV functional level and underwent transplantation evaluation. Considerations for lung transplantation included a recent history of biopsy-confirmed (2009 and 2011) hepatitis that included a broad differential of autoimmune, viral, and drug-induced disease etiologies on histopathology. Repeat liver biopsy in 2012, however, was most suggestive of autoimmune hepatitis given the substantial reduction in lobular lymphoid hyperplasia observed between 2011 and 2012 in response to a course of suppressive prednisone and azathioprine therapy. Of note, his pulmonary function continued to worsen during this time despite the immunosuppression.

**Figure 1:**
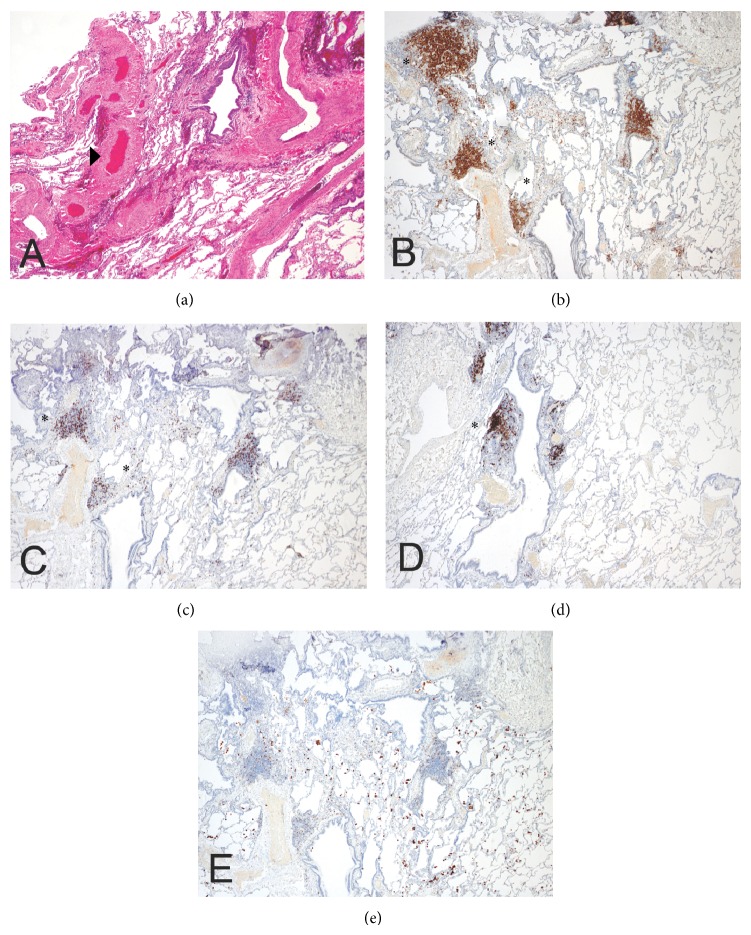
Small pulmonary AVMs (▸) with peripherally localizing lymphoid aggregates (∗) were confirmed on explanted lung pathology. Immunohistochemical staining directed against (a) H/E, (b) CD4, (c) CD8, (d) CD20, and (e) CD68.

The patient received bilateral cadaveric donor lung transplantation without requirement for cardiopulmonary bypass on November 21, 2012, at the age of 25. Following surgery, he received standard of care immunosuppression with three doses of antithymocyte globulin and two doses of high-dose methylprednisolone. He was subsequently placed on a steroid taper and started on tacrolimus (titrated to daily levels) and azathioprine (150 mg daily) with appropriate antiviral (donor CMV negative, recipient CMV positive), antibacterial, and antifungal prophylaxis. He was successfully extubated by postoperative day three. Repeat pulmonary function tests and ventilation/perfusion scan demonstrated excellent function of the transplanted organs without early concern for mechanical failure. He was discharged home on postoperative day 15. Subsequent analysis of explanted lung tissue confirmed the diagnosis of small pulmonary AVMs with peripherally localizing lymphoid nodules (Figure 1).

At the 21-month follow-up from transplantation, the patient continues on tacrolimus, prednisone, and sirolimus for immune suppression (switched from mycophenolate mofetil secondary to leukopenia). His posttransplantation course has been complicated by lymphocytic small airway inflammation and minimal acute rejection on serial graft biopsies. More recently (June of 2014), he began to develop accelerated bronchiolitis obliterans syndrome that is currently being managed with extracorporeal photopheresis. He additionally continues on IVIG infusions (40 g every three weeks), which are anticipated to continue life-long for immune protection.

## 3. Discussion

This is a case of confirmed autoimmune-mediated cytopenias and confirmed humoral immune deficiency, which does not meet criteria for CVID given the preserved level of total IgG (Table 1). Despite the splenomegaly, autoimmune-mediated cytopenias, and question of an elevated double-negative T cell population (Table 3), the patient screened negative for ALPS on gene sequencing and analysis and otherwise does not fit the full ALPS presentation given the preserved population of memory B cells, the defect in T cell proliferation, and the normal levels of total IgG and IgM in this case [7]. Therefore, we are left with a diagnosis of humoral immune deficiency, specific to IgA, IgG2, and IgG4. While the noted humoral deficits could certainly have predated the patient's splenectomy in 2005, this hypothesis was never validated on laboratory testing and certainly a description of recurrent infections is not clearly documented at this time in the patient's history. Alternatively, the humoral deficits could have developed subsequent to the patient's splenectomy. Overall, humoral immune deficiency is known to be associated with autoimmune-mediated cytopenias. In a retrospective chart review of 326 CVID patients [10], 11% had a history of autoimmune-mediated cytopenias, 54% of which had the first episode of thrombocytopenia or hemolytic anemia prior to the diagnosis of CVID. However, postsplenectomy immune deficiency has also been described. In an analysis of 12 patients who underwent traumatic splenectomy [11], decreased B cell activation was observed in response to polyclonal activator pokeweed mitogen at two days to seven years postoperatively. Coculture analysis further confirmed that the defect was associated with both the T helper cell and the intrinsic B cell compartment of the splenectomized patient. In a more recent comparative analysis of 209 asplenic or hyposplenic adults compared to 140 healthy controls [12], similar total levels of B cells were described. In comparison to the healthy controls, however, the splenectomized patients had a significant reduction (*P* < 0.001) in memory B cells, including both IgM memory B cells and switched memory B cells. Consistent with these data, here we describe preservation of total B cell counts, but with maturation arrest at the IgM+/IgD+ stage of development. Furthermore, genetic testing for known mutations linked to B cell maturation arrest was negative. Therefore, it remains uncertain as to whether the patient had a humoral immune deficiency prior to splenectomy or whether the splenectomy itself initiated a B cell dysregulation. Together these data highlight the importance of screening for humoral immune deficiencies concurrent with the diagnosis of autoimmune-mediated cytopenia and prior to splenectomy and initiation of the perisplenectomy vaccination series.

Pulmonary complications including granulomas, bronchiectasis, and emphysema are known to be associated with primary humoral immune deficiencies [6]. However, pulmonary AVMs are not a frequent complication of humoral immune deficiency, as described in chart [6] or even direct radiographic [13] review. To our knowledge, there are only two case reports linking humoral immune deficiency to pulmonary AVMs, described in a patient with hypogammaglobulinemia [14] as well as IgA and IgG subclass deficiency [15]. We also entertained the possibility that splenectomy itself could have driven the pulmonary pathology in this case. While postsplenectomy pulmonary hypertension driven by chronic thromboemboli has been described [16], our patient had no evidence of pulmonary hypertension on right heart catheterization in 2009, and furthermore pulmonary AVMs are not a known complication after splenectomy [17, 18]. Alternative diagnoses were entertained in consultation with the medical genetics team at Massachusetts General Hospital. However, the patient screened negative for the genetic mutations most frequently associated with HHT (*ACVRL1* and* ENG*). Furthermore, HHT is not commonly associated with the humoral immune deficiencies otherwise described in this case [19]. Given that the pulmonary AVMs were diffuse and small in nature, the patient was not an appropriate candidate for embolization, and he did not clinically benefit from the pulmonary wedge resection. Ultimately the explanted lung pathology confirmed prominent and diffuse AVMs with small surrounding lymphoid nodules (Figure 1). It is unclear if the lymphocytes were driving the vascular pathology in this case. However, the physiology is clear, remarkable for oxygen-resistant hypoxemia with worsening ventilation/perfusion mismatch as the driving etiology for transplantation.

Lung transplantation in patients with humoral immune deficiency is a rare therapeutic endpoint. Several case reports have been described in patients with panhypogammaglobulinemia for the indications of end-stage bronchiectasis, emphysema, and/or granulomatous disease [20–23]. Lung transplantation has also been described in a single patient with IgG1 subclass deficiency, who was transplanted for the indication of end-stage bronchiectasis and was still alive at the nine-year follow-up [24]. Case reports of lung transplantation for the indication of diffuse pulmonary AVMs have also been described. Of note, these case reports are limited to patients with a confirmed diagnosis of HHT [25–28]. Therefore, while it is unclear whether the immune deficiencies herein described predisposed the patient to pulmonary AVMs, this is a unique case description of bilateral lung transplantation for the indication of pulmonary AVMs in a patient without a confirmed diagnosis of HHT but with a confirmed diagnosis of humoral immune deficiency.

IVIG is an important component of posttransplantation management, as hypogammaglobulinemia can be observed in an immunologically competent patient in the context of immunosuppressive therapy. In solid organ transplantation, a significant reduction in risk for infection was recently described with IVIG replacement of approximately 500 mg/kg monthly [29]. In our patient, this calculates to 32 g of IVIG monthly, which is less than the actual 40 g of IVIG that he has been receiving every three weeks. However, much of the management of humoral immune deficiency, especially in the context of solid organ transplantation, remains elusive. For example, while an anti-infective role for IVIG in transplantation has been described [29], the immune-modulatory effects of IVIG are still only partially understood. Additionally, we lack the long-term follow-up necessary to investigate whether patients with humoral immune deficiency are at risk of developing recurrent disease in their transplanted organs.

A growing body of data demonstrates the benefit of hematopoietic stem cell transplantation for the treatment of primary immune deficiency. Survival and cure after transplantation in the context of a well-matched donor, limited infectious disease complications, and no known end-organ damage has reached 90% [30]. For CVID specifically, allogeneic stem cell transplantation was recently described in a cohort of four patients for the indication of malignancy (two patients) and severe end-organ failure with documented granulomatous disease (two patients) [31]. Three of the four patients had long-term survival (between 4.5 and 7 years). However, only one in four patients demonstrated sustained IgG normalization with adequate vaccine response after transplantation, suggesting a true cure for CVID. In patients with primary immune deficiency and end-stage lung disease, a current clinical trial is underway that will directly address whether bilateral orthotopic lung transplantation followed by cadaveric partially matched hematopoietic stem cell transplantation is safe and effective [32], a proof of principle case report having already been demonstrated in 2011 [33]. These data will play a critical epidemiologic role, furthering our understanding of cotransplantation risk and long-term benefit and ultimately directing superior management of humoral immune deficiency and its pulmonary complications.

## Figures and Tables

**Table 1:** The patient had confirmed deficiencies in IgA, IgG2, and IgG4. Immunoglobulin levels are expressed in mg/dL with age-matched reference ranges provided.

	2005	2011	2012
On IVIG	No	Yes	Yes
Trough level	No	Yes	Yes
Low albumin	No	Yes	No

IgG	1240 (639–1344 mg/dL)	2540∗∗ (767–1590 mg/dL)	1230 (614–1295 mg/dL)
IgG1	960 (422–1292 mg/dL)	1730∗∗ (341–894 mg/dL)	
IgG2	14∗ (117–747 mg/dL)	171 (171–632 mg/dL)	
IgG3	275∗∗ (41–129 mg/dL)	299∗∗ (18–106 mg/dL)	
IgG4	5∗ (10–67 mg/dL)	13 (2–121 mg/dL)	
IgA	11∗ (70–312 mg/dL)	<7∗ (69–309 mg/dL)	<7∗ (69–309 mg/dL)
IgM	151 (34–210 mg/dL)	591∗∗ (53–334 mg/dL)	125 (53–334 mg/dL)
IgE		6 (0–100 mg/dL)	

^*^Abnormally low levels. ^**^Abnormally high levels. Data are presented from 2005 (before IVIG therapy), 2011 (trough level during IVIG therapy at a time of hypoalbuminemia), and 2012 (trough level during IVIG therapy at a time of normal serum albumin).

**Table 2:** The patient had confirmed anemia and neutropenia. Complete blood counts from 2009 expressed as a range and a median with reference ranges provided.

	Range	Median	Reference
WBC	3.7–9.8	5.2	4.5–11.0 th/*μ*L
HGB	9.7–12.0∗	10.2∗	13.5–17.5 g/dL
HCT	30.6–37.3∗	32.2∗	41.0–53.0%
PLT	259–385	308	150–400 th/*μ*L
Neutrophils	0.00–1.48∗	0.69∗	1.80–7.70 th/*μ*L
Lymphocytes	1.48–5.78	2.20	1.00–4.80 th/*μ*L
Monocytes	1.03–3.63	1.32	0.20–1.20 th/*μ*L
Eosinophils	0.00–0.77	0.40	0.00–0.90 th/*μ*L
Basophils	0.00–0.42	0.10	0.00–0.30 th/*μ*L

^*^Abnormally low levels. White blood cells (WBC), hemoglobin (HGB), hematocrit (HCT), and platelets (PLT) are shown.

**Table 3:** The patient had preserved populations of total T cells, T cell subsets, total B cells, and memory B cells. There was question of an elevated double-negative T cell population that was not confirmed on direct TCR*αβ* staining. Flow cytometry analysis of peripheral blood cells in 2009.

	Major antigen	Minor antigen	Measured (cells/mm^3^)	Reference range (cells/mm^3^)	% of total	% of CD3+	% of CD19+
T cells	CD3+		4092	690–2540	86%	100%	
	CD3+	CD4+	3072	419–1590	64%	75%	
	CD3+	CD8+	785	190–1140	16%	19%	
	CD3+	CD4−/CD8−	235		5%	6%	

B cells	CD19+		352	90–660	7%		100%
	CD19+	CD27+	107		2%		30%
	CD19+	CD27−	245		5%		70%

NK cells	CD16+/56+		335	90–590	7%		

Data are presented in cells/mm^3^ or % with reference ranges provided (note that reference ranges for CD4−/CD8− cells, CD27+ cells, and CD27− cells are not established at the Massachusetts General Hospital, and thus normal was defined according to percentages previously described [7, 8]).
